# The role of socio-cultural-demographics in long-term haemodialysis survival

**DOI:** 10.6026/973206300210606

**Published:** 2025-04-30

**Authors:** Siddharth Garg, Mohamad Akram, Shahbaj Ahmad

**Affiliations:** 1 Department of Medicine, Himalayan Institute of Medical Sciences, Swami Rama Himalayan University, Jolly Grant, Dehradun, India

**Keywords:** Chronic kidney disease, long-term haemodialysis, socio-cultural-demographic, blood pressure

## Abstract

The socio-cultural-demographic attributes of long-term haemodialysis (HD) treatment survival is of interest. Hence, 129 adult chronic kidney disease patients, who have been on maintenance haemodialysis for >3 years, were evaluated for socio-economic demographics traits, clinical parameters and laboratory investigations. The most prevalent symptoms were loss of appetite (76.0%); anaemia (67.4%) and pedal edema (57.4%) with most patients underwent dialysis 2-3 times per week (49.6%). Vascular access was primarily through arteriovenous fistulas (55.8%); nephrologist supervision was present for 93.0% of patients with 62% patients had mild (10 - 11.9g/dL) anaemia. Age, gender, comorbidities, frequency of dialysis, duration of haemodialysis, nephrologist supervision and patients' travel distance emerged as significant factors for their association with long-term haemodialysis.

## Background:

Being a recognized global health burden, chronic kidney disease (CKD), affects approximately 10-15% of the general adult population in high and low-income countries [1]. This debilitating illness impacts close to 7% adults aged 30 years and older (70 million people) [2]; although this number might well be an underestimation given the stepping burden of undiagnosed chronic kidney disease in underdeveloped countries as its prevalence is likely to rise further considering the staggering progression in co-morbidities such diabetes, hypertension and obesity, in elderly population [3]. Chronic kidney disease is characterized by progressive and irreversible loss of kidney function, defined as either: 1. the presence of kidney damage or 2. A glomerular filtration rate (GFR) of less than 60 ml/min for three months or longer. The progressive loss of renal function, subsequently, leads to end-stage renal disease (ESRD) that essentially requires renal replacement therapy (RRT): a renal transplant or haemodialysis (HD) [4]. Hemodialysis despite being an effective therapeutic measure is costly and outside the affordability realm of majority of the patients [5]. Despite the end-outcome of chronic kidney disease maybe death - the interim lifespan - could be ameliorated via subjecting patients to RRT [6]. Nonetheless, the long-term survival rate of patients undergoing hemodialysis, the more preferred treatment modality is determined by many factors pertinent to the patient himself, his familial attributes (including socio-demographic variables) and how their bodies respond to the disease [7]. Currently, maintenance haemodialysis (MHD) is the preferred choice of RTT both by patients and their associates. MHD, despite being perceived as an RTT variant (in chronic kidney disease patients) meant to support life; there are multiple factors which play a role in deciding its over-all effectiveness [8]. Notably, chronic kidney disease patients have a myriad of life-long restrictions, viz., change in daily lifestyles, physical limitations, social restrictions, dependency on regular follow ups, decreased libido, emotional distress *etc.* to name a few. Not only hemodialysis prolongs survival and reduces morbidities, but also improves the quality of life, overall [6].

Common independent predictors of survival are age, race, serum albumin (at the start of dialysis), activity level at the start of dialysis and presence of co-morbidities (*e.g.*, heat failure and cancer). Indeed, patient survival was better with higher dialysis dose, increased frequency of dialysis and adequate serum albumin level [9]. Additionally, efforts at minimizing complications from infectious diseases, preventing cardiovascular events and better nutrition have shown to improve survival rates among haemodialysis patients [10]. One key attribute to long-term survival was patient's ability to adapt to the treatment; specifically these are highly motivated individuals who owe to maintain their health and adhere to the complex dietary/medication requirements of hemodialysis treatment [11]. They are often seen actively involved in their care, participating in decisions related to their treatment, working closely with their healthcare team [12]. As such patients have often experienced significant challenges related to their illness, including physical and emotional symptoms, financial difficulties and social isolation; yet they maintain a positive outlook and continue to engage in activities they enjoy [13].

Long-term surviving hemodialysis patients may also have specific clinical characteristics that contribute to their longevity. These may include lower levels of inflammation and oxidative stress, as well as better control of blood pressure, diabetes or heart disease, which can contribute to poor outcomes in hemodialysis patients [14]. Finally, long-term surviving hemodialysis patients may have access to specialized renal-care and support which may include but not limited to patient support groups and access to advanced care options, specifically high-quality hemodialysis treatment, regular monitoring of their treatment parameters, adequate dialysis dose and proper infection control measures [15]. Additionally, such patients may also receive better: nutritional status, adherence to dietary restrictions. *i.e.*, meagre protein/potassium intake, circumvent complications such as hyperkalaemia and malnutrition alongside specialized nutritional support, such as vitamin and mineral supplementation [16]. Patients undergoing maintenance haemodialysis have had a high rate of mortality rate and they require complex care throughout [17]. As their illness trajectories are variable, it is imperative to mirror predict their prognosis to provide quality care. Notably, despite patients' desire to know their prognosis, evidence suggests that this information is not commonly shared and often results in high treatment intensity at the end of life, not aligned with patients' preferences [18]. While prognostic systems for dialysis patients exist, few studies have combined prognostic factors into clinically useful prediction tools, with most focusing on incident patients. Current models for predicting death have limited external validity and none have been implemented and evaluated in clinical practice on a large scale [19]. The development of prognostic models that can be integrated at the bedside to guide clinical care is a priority in nephrology and can be included in different strategic plans [20]. However, to date no studies have evaluated the performance or usefulness of prognostic tools in real-life clinical practice for prevalent haemodialysis patients. Therefore, it is of interest to study: (i) the social-economical demographic data in context of the long- term survival in patients undergoing haemodialysis and (ii) the various socio-cultural-demographic attributes of haemodialysis patient under the unified lens of long-term survival.

## Methods:

## Participants:

One hundred and twenty-nine adult chronic kidney disease patients attending the Dialysis Unit of Himalayan Institute of Medical Sciences Hospital, who have been on maintenance hemodialysis for >3 years were recruited. However, patients are not on life-long haemodialysis or suffering from terminal illness from chronic kidney disease (malignancies) and/or with mental debility and psychiatric illnesses. Ethical approval/clearance from the Institutional Ethics Committee which conformed to the journal standards was granted prior to recruitment. The data collection for this study was conducted in accordance with the Declaration of Helsinki Declaration of Helsinki (REC Approval Code: SRHU/HIMS/RC/2024/274).

## Experimental protocol:

All patients diagnosed with chronic kidney disease and attending the Dialysis Unit were briefly enquired about the duration of illness and dialysis and those undergoing Haemodialysis for more than 3 years were recruited as per the inclusion/exclusion criteria stated above. Once, consented they were taken for further evaluation alongside history taking. Subsequently, the socio-economic demographics, clinical parameters and laboratory investigations (detailed below) were recorded.

A data collection sheet, split into the two sections: Section A: Socio-economical-demographic attributes and 2. Section B: Clinical characteristics of subjects including short history and lab investigations was used to collect patient data (ANNEXURE - 1) Specifically, Section A: age, gender, marital status, familial status (joint family/ unitary), educational status. Section B: Clinical signs/symptoms: loss of weight, loss of appetite, fever, leg cramps, breathlessness, bleeding from any site, jaundice, itching/ dermatopathy, impaired cognitive function, anaemia, pedal edema, palpitations; comorbidities: hypertension, diabetes mellitus, coronary artery disease; treatment factors: frequency of dialysis, duration, vascular access, nephrologist supervision, patients' travel distance from dialysis centre. The recorded lab investigations included haemoglobin, iron studies, potassium/sodium/ bicarbonate over long term, serum albumin, residual urine output and miscellaneous investigations as per need (Calcium/Phosphorus/PTH/25 Hydroxy Vitamin D/ X-ray LS Spine/2 D Echo).

## Sample size calculation:

The following simple formula was used for calculating the adequate sample size: n= (Z1-α/2) 2P (1-P)/d2, where n is the sample size, Z is the standard normal deviate for alpha error of 5% and 2-sided, 2-tailed analysis 95% confidence level = 1.96, P is expected prevalence (previous literature shows prevalence of chronic kidney disease amongst general population = 0.14) and d is precision. (The precision has been taken as 6 in my study). N = 1.96 x 1.96 x 0.14 (1-0.14)/ (0.06*0.06) = 4625/3 = ~129.

## Statistical analysis:

The data was collected tin MS Excel (MS Office t2010) and tabulated. SPSS Software version t25 was used to analyse the data that has been tabulated. Descriptive statistics like frequency charts were used for qualitative variables. An association between the various factors and long-term survival was established using ta Chi Square Test tor ta Fischer Exact Test. Other tests like scatter plots, *etc.* were used as per applicability. Quantitative data was expressed using Mean t± SD. A p value of t< t0.05 was considered significant.

## Results:

In Table 1, the majority of patients were within the age range of 30 to 50 years (49.6%), with 17.1% below 30 years and 33.3% over 50 years old. Sex distribution showed 55.8% male and 44.2% female patients with 66.7% were married, while 33.3% were unmarried. Familial status indicated that 30.2% belonged to joint families, whereas 69.8% lived in unitary households. Educational status varied, with 34.9% having a high school education, 44.2% being college graduates and 20.9% holding postgraduate degrees. The most prevalent symptoms reported among the long-term hemodialysis patients included loss of appetite (76.0%), anaemia (67.4%) and pedal edema (57.4%). Other symptoms were loss of weight (33.3%), itching/dermatopathy (40.3%), fever (47.3%) and breathlessness (24.8%). Hypertension was the most common comorbidity, affecting 73.6% of patients, followed by diabetes mellitus, which was present in 48.8% of cases. A smaller proportion, 21.7%, had a history of coronary artery disease. In Table 2, the majority underwent dialysis 2-3 times per week (49.6%), with smaller proportions receiving dialysis less frequently (3 times per week, 41.1%). Whilst the highest percentage of patients fell within the 3-5-year range (34.9%), this was followed by 5-7 years (28.7%), 7-10 years (18.6%) and over 10 years (17.8%). Vascular access for dialysis was primarily through arteriovenous fistulas (55.8%), while central venous catheters (24.8%) and AV grafts (19.4%) were less common. Nephrologist supervision was widely prevalent with around 93.0% of patients receiving care under their supervision. Finally, patients' travel distance from the dialysis centre varied, with 52.7% living within 10 km, 27.1% residing 10-20 km away and 20.2% having to travel over 20 km.

In Table 3, the mean haemoglobin level is 11.7 ± 5.7 g/dL with 80 patients had mild (10 - 11.9g/dL) and 29 patients had moderate anaemia (7 - 9.9g/dL). For serum iron levels: 17 patients had levels below 50 µg/dL, 32 patients fell within the range of 50 to 60 µg/dL, 24 patients had levels between 60 and 70 µg/dL, 52 27 patients had levels between 70 and 80 µg/dL, 21 patients had levels between 80 and 90 µg/dL and 8 patients had iron levels above 90 µg/dL. For the total iron-binding capacity (TIBC): 13 patients had TIBC levels below 200 µg/dL, 36 patients fell within the range of 200 to 220 µg/dL, 24 patients had levels between 220 and 240 µg/dL, 32 patients had levels between 240 and 260 µg/dL, 18 patients had levels between 260 and 280 µg/dL and 6 patients had TIBC levels above 280 µg/dL. For serum albumin levels: 12 patients had levels less than 3.3 g/dL, 18 patients fell within the range of 3.3 to 3.5 g/dL, 25 patients had levels between 3.5 and 3.7 g/dL, 30 patients had levels between 3.7 and 3.9 g/dL, 23 patients had levels between 3.9 and 4.1 g/dL, 14 patients had levels between 4.1 and 4.3 g/dL, 7 patients had levels between 4.3 and 4.5 g/dL and none had albumin levels above 4.5 g/dL. Per the residual urine output: 6 patients had levels below 100 mL/day, 14 patients had levels between 100 and 200 mL/day, 24 patients had levels between 200 and 300 mL/day, 30 patients had levels between 300 and 400 mL/day and 55 patients had levels between 400 and 500 mL/day. The mean calcium, phosphorus, PTH and 25- Hydroxy Vitamin D were 9.2 ± 4.1 mg/dL, 3.3 ± 1.8 mg/dL, 43 ± 25.6 pg/mL and 24.8 ± 11.3 ng/mL respectively with most patients had normal levels of calcium, phosphorus and PTH.

In Table 4, for the 3-5 years survival group, 10 patients were below 30 years old, 25 were between 30 and 50 years old and 10 were above 50 years old. In the 5-7 years survival group, there were 6 patients below 30 years old, 20 patients aged between 30 and 50 and 11 patients above 50 years old. In the 7-10 years survival group, 4 patients were below 30 years old, 12 were between 30 and 50 years old and 8 were above 50 years old. In the > 10 years survival group, 2 patients were below 30 years old, 7 were between 30 and 50 years old and 14 were above 50 years old. There was no statistically significant association between age and survival group of the patients (p >0.05). There was statistically significant association of gender with long term survival (p 10 years as compared to males. None of the comorbidities (hypertension, diabetes mellitus, or c there was no statistically significant association between the frequency of dialysis and long-term survival for the patient population studied. Coronary artery disease) showed a statistically significant association with long term survival. Also, it shows how patients in different survival groups (3-5 years, 5-7 years, 7-10 years and more than 10 years) were distributed across various durations of hemodialysis. The p value is highly significant (P < 0.001), indicating a strong statistical association between the duration of hemodialysis and long-term survival. It suggests that patients who have been on hemodialysis for a longer duration are more likely to survive longer as compared to those with shorter durations of hemodialysis. There was no statistically significant association between the type of vascular access and long-term survival for the patient population studied (p >0.05). There was strong statistical association between nephrologist supervision and long-term survival (p < 0.001) indicating that those patients whose hemodialysis was conducted under the supervision of nephrologist were more likely to survive longer. Lastly, a strong statistical association was observed between patients' travel distance and their longterm survival (p < 0.001). Patients who were living within a distance of 10 km from the hemodialysis facility survived longer as compared to those who were living far away.

## Discussion:

We were interested to study: (i) the social-economical demographic data in context of the long- term survival in patients undergoing haemodialysis and (ii) the various attributes of haemodialysis patient under the unified lens of long-term survival. For Aim (i), we found that the majority of patients were between 30 to 50 years old, males, married, lived in unitary households and had varying levels of educational attainment. Hypertension was the most common comorbidity. Treatment factors such as dialysis frequency, duration of haemodialysis, vascular access type, nephrologist supervision and patients' travel distance from the dialysis centre were examined. Notably, the majority of patients underwent dialysis 2-3 times per week, with arteriovenous fistula being the primary vascular access method. For Aim (ii), age, gender, comorbidities, frequency of dialysis, duration of haemodialysis, nephrologist supervision and patients' travel distance emerged as significant factors pertinent to their association with long-term survival. The results of the present study align closely with prior research conducted by Singh *et al.* [21] and Rubio *et al.* [22]. In both the studies, a demographic pattern emerged. The majority of participants in these studies fall within the age range of 30 to 50 years (49.6%), with 17.1% below 30 years and 33.3% over 50 years old. Gender distribution consistently shows 55.8% male and 44.2% female participants with 66.7% being married and 33.3% unmarried in the present study. 30.2% belonged to joint families, while 69.8% lived in unitary households. Educational status exhibits variations, with 34.9% having a high school education, 44.2% being college graduates and 20.9% holding postgraduate degrees across all studies. This congruence in demographic patterns strengthens the reliability and generalizability of the findings, providing a robust foundation for further analysis and interpretation. Hypertension is reported in 95 cases, constituting a 73.6% of the studied population. Diabetes Mellitus is observed in 63 (48.8%) and coronary artery disease is identified in 28 (21.7%). Importantly, the survey allowed for multiple responses, emphasizing the intricate nature of comorbid conditions in this patient group. Our results align with studies conducted by Gallieni *et al.* [23] and Chen *et al.* [24]; hypertension emerges as the most prevalent comorbidity closely followed by diabetes mellitus. Additionally, 21.7% have had a history of coronary artery disease. The consistent prevalence of these comorbidities across different studies highlights their substantial impact on the hemodialysis patient population and underscores the need for targeted interventions addressing these common health challenges. In our study, 49.6% of patients undergoing treatment 2 times per week and 41.1% opting for a thrice-weekly 70 schedule, resulting in a mean of 2.3 ± 1.6 sessions per week. The duration of hemodialysis highlights a predominant portion falling within the 3-5 years category (34.9%), followed by those with 5-7 years of treatment (28.7%). Vascular access preferences underscore a significant inclination toward arteriovenous-fistula (55.8%), while central venous catheter (24.8%) and AV Graft (19.4%) are less frequently chosen. In our study, the highest percentage of patients falling within the 3-5-year range (34.9%), followed by 5-7 years (28.7%), 7-10 years (18.6%) and over 10 years (17.8%). Vascular access for dialysis exhibited a similar pattern, predominantly through arteriovenous fistulas (55.8%), while central 71 venous catheters (24.8%) and AV grafts (19.4%) were less common,. Nephrologist supervision was observed in 93.0% of our patients receiving care, aligning closely with the reported figures. Lastly, patients' travel distances from the dialysis center displayed comparable variability, with 52.7% residing. Our nuanced analysis provides a comprehensive perspective on the distribution of Serum Albumin levels among the study participants, shedding light on specific ranges that warrant attention and potential interventions. Notably, the findings of this study align closely with those of Anupama and Uma [25], indicating a similarity in the observed lower levels of serum albumin, emphasizing the need for further exploration and potential interventions in this regard.

The findings of the current study unveil a pronounced association between the duration of hemodialysis and the distribution of patients across distinct time intervals. Notably, participants undergoing hemodialysis for 3-5 years exclusively constituted the entirety of this duration category, with a conspicuous absence in the other duration groups (5-7 years, 7-10 years and >10 years). Subjected to hemodialysis for 5-7 years exhibited a similar exclusive pattern, accounting for 100.0% representation solely within this duration category, with no presence in the alternative groups. This consistent trend persists for participants falling within the 7-10 years and >10 years duration categories, with a complete 100.0% representation in their respective groups and an absence in the remaining ones. The robust statistical analysis, as reflected by the p-value (<0.001), accentuates the significance of these discernible patterns, underscoring a clear association between the duration of hemodialysis and the specific distribution of participants across the delineated time intervals. Importantly, these results align with a study conducted by Mapes *et al.* [26], validating the consistency of the observed associations in the broader context of hemodialysis duration and participant distribution. The findings of the present study unveil a consistent trend in the utilization of vascular access types among hemodialysis patients with varying treatment durations. Arteriovenous fistula (AVF) emerges as the predominant choice across all observed groups, constituting a substantial percentage ranging from 77.8% to 87.5%. In contrast, the use of central venous catheters (CVC) remains consistently low. Statistical analysis reveals no significant differences in the distribution of vascular access types across various time intervals. This implies that the preference for a specific vascular access type remains relatively stable regardless of the duration of hemodialysis treatment. Noteworthy is a marginal increase in the utilization of AV grafts (4.3%) among patients with a treatment duration exceeding 10 years, suggesting a potential shift in access preferences over extended treatment periods. Importantly, it is worth mentioning that these results diverge from those reported in a study conducted by Hays *et al.* [27], indicating potential variations in vascular access patterns across different research contexts. The findings of the current study illuminate a significant association between Nephrologist Supervision and the duration of hemodialysis, underscored by a noteworthy p-value of <0.001. In the cohort undergoing hemodialysis for 3-5 years (n=45), a striking 100.0% reported Nephrologist Supervision and none indicated the absence of such supervision. Similarly, for patients in the 5-7 years (n=37) and 7-10 years (n=24) duration categories, all individuals (100.0%) received Nephrologist Supervision, with none reporting otherwise. Notably, within the group with a hemodialysis duration exceeding 10 years (n=23), a statistically significant distinction emerges. Here, 60.9% reported Nephrologist Supervision, while 39.1% indicated no supervision, a difference deemed statistically significant (p<0.001). This observation accentuates the potential influence of prolonged hemodialysis duration on 83 the preference for Nephrologist Supervision, prompting a consideration for tailored care strategies in scenarios of extended hemodialysis. It is noteworthy that these results align with those reported in a study conducted by Mani [28], emphasizing the consistency of the findings across different investigations. The findings from the current investigation underscore a noteworthy and statistically significant association between patients' travel distance and the duration of hemodialysis treatment. Specifically, for patients residing within a travel distance of less than 10 km, a considerable 66.7% had been undergoing hemodialysis for 3-5 years. In contrast, within the 10-20 km range, a substantial 70.3% of patients exhibited treatment duration of 5-7 years. Notably, among patients traveling more than 20 km, a striking 82.6% had been on hemodialysis for over 10 years. The observed distribution across these distinct travel distances demonstrated a high level of significance (p < 0.001), indicating a robust correlation. This significant association suggests that travel distance might exert a crucial influence on the long-term adherence and persistence of patients in hemodialysis treatment, with those residing farther experiencing a higher longevity in treatment duration. It is noteworthy that these results diverge from those reported in the study conducted by Masina *et al.* [29], highlighting the importance of considering contextual factors and variations in healthcare settings related to travel distance and hemodialysis treatment duration. Although not directly assessed; however, socio-economic, environmental and lifestyles factors also play a significant role in determining haemodialysis survival rates as one-, two- and three-year as Hekmat *et al.* reported lower one-, two- and three-year survival in Afghan patients compared to their Iranian and Japanese peers [30], as suggested by the Japanese Society for Dialysis Therapy (JSDT) and Okinawa Dialysis Study (OKIDS) data [31]. To take our findings and the current long-term hemodialysis landscape forward, there is an urgent need to develop dialysis modalities that are cost-effective, accessible and offer improved patient outcomes. While patients want longevity, reduce symptoms burden and attain maximal functional and social rehabilitation, it is critical for policymakers and healthcare systems to get on the same page as closely as possible.

## Conclusion:

The key attributes that affect long-term survival of haemodialysis patients include comorbidities, symptoms and treatment factors. Notably, the majority of patients underwent dialysis 2-3 times per week, with arteriovenous fistula being the primary vascular access method. The findings emphasize the importance of tailored treatment strategies, multidisciplinary care and patient-centred approaches to improve outcomes and enhance quality of life.

## Figures and Tables

**Table 1:** Demographic characteristics and clinical profile of the participants

**Demographic Characteristics**	**Frequency**				**Percentage (%)**
Age (years)	5-Mar	7-May	10-Jul	>10	
<30	10	6	4	2	
30-50	25	20	12	7	
>50	10	11	8	14	
Sex					
Male	30	25	10	7	
Female	15	12	14	16	
**Familial status**					
Joint family	11	19	6	4	
Nuclear	34	18	18	19	
Familial status					
High school	12	12	11	9	
Graduate	21	14	9	13	
Postgraduate	12	10	4	1	
**Symptoms**					
Loss of weight	43				33.3
Loss of appetite	98				76
Fever	61				47.3
Leg cramps	37				28.7
Breathlessness	32				24.8
Bleeding	19				14.7
Jaundice	25				19.4
Itching	52				40.3
Impaired Cognitive function	30				23.3
Anemia	87				64.7
Pedal Oedema	74				57.4
Palpitations	43				33.3
History of diabetes	20	18	10	15	
History of CAD	10	7	5	6	
History of systemic hypertension	35	25	15	20	

**Table 2:** Haemodialysis treatment profile

**Treatment Factors**	**Frequency**	**Percentage (%)**
**Frequency of Dialysis**		
1/week	12	9.3
2/week	64	49.6
3/week	53	41.1
**Nephrologist Supervision**		
Yes	120	93
No	9	7
**Duration of Hemodialysis**		
3-5	45	34.9
5-7	37	28.7
7-10	24	18.6
>10	23	17.8
**History of smoking**		
Arteriovenous Fistula	2	1.6
Central Venous Catheter	67	51.9
AV Graft	60	46.5
**Patients' Travel Distance from Dialysis Centre**		
<10 km	68	52.7
10-20 km	35	27.1
>20 km	26	20.2

**Table 3:** Clinical and biochemical treatment profile of the participants

**Treatment Factors**	**Frequency**	**Percentage (%)**
**Haemoglobin Level (g/dL)**		
≥ 12 (Normal)	20	15.5
10 - 11.9 (Mild anaemia)	80	62
7 - 9.9 (Moderate anaemia)	29	22.5
<7 (Severe anaemia)	0	0
**Serum Iron (µg/dL)**		
< 50	17	32.2
50-60	32	24.8
60-70	24	18.6
70-80	27	20.9
80-90	8	6.2
>90		
**Total Iron Binding Capacity (µg/dL)**		
< 200	13	10.1
200-220	36	27.9
220-240	24	18.6
240-260	32	24.8
260-280	18	13.9
>280	6	4.7
**Serum Albumin (g/dL)**		
< 3.3	12	9.3
3.3-3.5	18	13.9
3.5-3.7	25	19.4
3.7-3.9	30	23.3
3.9-4.1	23	17.9
4.1-4.3	14	10.8
4.3-3.5	7	5.4
>4.5	0	0
**Reduced Urine Output**		
< 100	6	4.7
100-200	14	10.9
200-300	24	18.6
300-400	30	23.2
400-500	55	42.6
**Calcium (mg/dL)**		
<8.5	14	10.8
8.5-10.4	90	69.8
>4.5	25	19.4
**Phosphorus (mg/dL)**		
<3.0	32	24.8
3.0-4.5	85	65.9
>4.5	12	9.3
**PTH (pg/mL)**		
<10	26	20.1
Oct-55	80	62
>55	23	17..9
**25 Hydroxy Vitamin D (ng/mL)**		
<20	38	29.4
20-30	73	56.6
>30	18	14

**Table 4:** Demographic comparison of the participants undergoing haemodialysis

**Age (years)**	**5-Mar**	**7-May**	**10-Jul**	**>10**	**p' value**
<30	10(22.2)	6(16.2)	4(16.7)	2(8.7)	
30-50	25(55.6)	20(54.1)	12(50)	7(30.4)	>0.05
>50	10(22.2)	11(29.7)	8(33.3)	14(60.8)	
**Sex**					
Male	30(66.7)	25(67.6)	10(41.7)	7(30.4)	<0.05
Female	15(33.3)	12(32.4)	14(58.3)	16(69.6)	
**Comorbidities**					
Diabetes Mellitus	20(44.4)	18(46.6)	10(41.7)	15(65.2)	>0.05
Hypertension	35(77.7)	25(67.6)	15(62.5)	20(87)	>0.05
CAD	10(22.2)	7(18.9)	5(20.8)	6(26.1)	>0.05
**Frequency of dialysis**					
<2/week	2(4.4)	5(13.5)	3(12.5)	2(8.7)	
2-3/week	18(40)	20(54.1)	12(50)	14(60.9)	>0.05
>3/week	25(55.6)	12(32.4)	9(35.7)	7(30.4)	
**Duration of hemodialysis**					
5-Mar	45(100)	0(0)	0(0)	0(0)	
7-May	0(0)	37(100)	0(0)	0(0)	<0.001
10-Jul	0(0)	0(0)	24(100)	0(0)	
>10	0(0)	0(0)	0(0)	23(0)	
**Vascular Access**					
Arteriovenous Fistula	35(77.8)	30(81.1)	21(87.5)	20(87.7)	
Central Venous Catheter	10(22.2)	7(18.9)	3(12.5)	2(8.7)	>0.05
AV Graft	0(0)	0(0)	0(0)	1(4.3)	
**Nephrologist Supervision**					
Yes	45(100)	37(100)	24(100)	14(60.9)	
No	0(0)	0(0)	0(0)	9(39.1)	
**Patients' Travel Distance from Dialysis Centre**					
<10 km	30(66.7)	26(70.3)	11(45.8)	1(4.3)	
10-20 km	12(26.7)	9(24.3)	11(45.8)	3(13.0)	<0.001
>20 km	3(6.7)	2(5.4)	2(8.3)	19(82.6)	
CAD: Coronary artery disease; AV Graft: Arterio-venous Graft
